# Fear of Missing Out in Academic Workers: A Theoretical Framework

**DOI:** 10.1111/nyas.70338

**Published:** 2026-07-14

**Authors:** Mattia Bozzetti, Alessio Lo Cascio, Daniele Napolitano

**Affiliations:** ^1^ Direction of Health Professions ASST Cremona Cremona Italy; ^2^ Direction of Health Professions La Maddalena Cancer Center Palermo Italy; ^3^ SITRA and Scientific Direction Fondazione Policlinico Gemelli IRCCS Rome Italy

**Keywords:** academic well‐being, engagement, fear of missing out, framework, self‐determination

## Abstract

This paper proposes the middle‐range theory “academic fear of missing out” (academic FoMO) as a situational and dynamic phenomenon emerging within competitive academic environments. Drawing on self‐determination theory, the model defines academic FoMO as a response to visibility pressure, motivational frustration, and perceived professional exclusion. Key constructs include overengagement, digital hypervigilance, and performance‐driven relational anxiety. The theory outlines antecedents, attributes, and psychological and behavioral consequences, particularly among early‐career and structurally vulnerable researchers. This framework aims to support empirical investigation and guide interventions to promote healthier academic cultures in the nursing and health professions.

## Graphical abstract

The present paper undertakes a theory‐informed concept analysis of a particular form of apprehension we term “academic fear of missing out” (FoMO) that is oriented toward missed opportunities, visibility, and professional inclusion and arises within environments marked by need frustration, intensified social comparison, and structurally embedded performance demands.

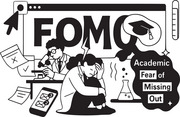

## 1 Introduction

Fear of missing out (FoMO) has been defined as a “pervasive apprehension that others might be having rewarding experiences from which one is absent” [1]. Although originally examined in adolescent and social media contexts, FoMO has increasingly been conceptualized as a broader motivational–affective response to perceived exclusion from valued experiences and opportunities. Within self‐determination theory (SDT), FoMO has been linked to the frustration of basic psychological needs for autonomy, competence, and relatedness, suggesting that it may emerge when individuals perceive diminished control, reduced efficacy, or weakened social integration [1, 2, 3]. In this sense, FoMO reflects not merely a situational concern, but a regulatory signal associated with unmet psychological needs and heightened sensitivity to social comparison and belongingness threats.

While much of the empirical literature has focused on digital and social media behavior, a growing body of evidence indicates that FoMO is associated with lower well‐being, higher stress and negative affect, and compulsive monitoring behaviors, and that these patterns extend beyond leisure contexts [1, 4, 5, 6]. Importantly, recent research has begun to document the relevance of FoMO in occupational settings. For instance, workplace FoMO has been shown to predict burnout, message‐checking behavior, and work‐related strain, suggesting that FoMO may represent a meaningful mechanism linking environmental demands to maladaptive patterns of engagement at work [7]. These findings collectively support the view that FoMO is not inherently tied to specific domains (e.g., social media), but may emerge in contexts characterized by uncertainty, evaluative comparison, and pressure to remain continuously informed and responsive [8].

Contemporary academic environments display many of these characteristics. Academic careers are increasingly shaped by metric‐based evaluation systems, publication pressure, competitive funding structures, and precarious employment trajectories, particularly among early‐career researchers [9, 10, 11]. At the same time, the visibility of others’ achievements—through publications, grants, conferences, and digital platforms—amplifies opportunities for upward social comparison and perceived exclusion [12]. Within such environments, maintaining visibility and responsiveness may become central to professional identity and career progression, thereby increasing sensitivity to missed opportunities and signals of noninclusion.

Despite these converging conditions, the motivational and relational dynamics that may link academic work environments to patterns of vigilance, overengagement, and psychological strain remain insufficiently theorized. Existing constructs capture important aspects of academic work but do not fully account for the anticipatory apprehension of missing professionally valued opportunities [8]. For example, publication pressure focuses on stress associated with publishing demands [9], impostor phenomenon captures self‐doubt and fear of intellectual inadequacy, workaholism and overcommitment describe excessive work investment [17], and telepressure concerns the perceived need for rapid responsiveness to communication [13]. While conceptually related, these constructs do not explicitly address the fear of exclusion from opportunity structures and visibility circuits that may drive continuous monitoring and engagement behaviors.

From this perspective, FoMO may assume a domain‐specific configuration within academic work. The present paper therefore undertakes a theory‐informed concept analysis of a particular form of apprehension oriented toward missed opportunities, visibility, and professional inclusion, arising within environments marked by need frustration, intensified social comparison, and structurally embedded performance demands. Rather than introducing a generic new label for academic strain, the analysis seeks to clarify whether academic FoMO constitutes a conceptually coherent pattern with recognizable antecedents, defining attributes, and consequences. In doing so, it aims to provide a more precise conceptual basis for subsequent empirical inquiry in academic and health‐professions settings [14].

## 2 Conceptual Approach

The present manuscript adopts a theory‐informed concept analysis to clarify the structure of academic FoMO. Guided by Walker and Avant [15], the analysis focused on the elements that help specify what the concept is (defining attributes), what tends to precede it (antecedents), and what tends to follow from it (consequences). Defining attributes were treated as recurring characteristics whose absence would alter the nature of the phenomenon, whereas antecedents and consequences were retained only when they plausibly preceded or followed the activation of academic FoMO in a systematic and conceptually meaningful way.

In selecting these elements, we prioritized factors that (1) showed convergence across the broader FoMO literature, including both general and workplace contexts; (2) were theoretically consistent with the multilevel framework adopted (e.g., need frustration, social comparison, and structurally embedded opportunity asymmetries); and (3) contributed directly to explaining the emergence or maintenance of FoMO‐related responses. Conversely, factors that were more distal, highly context‐specific, or only weakly connected to the core apprehension of missing out were not retained in the central model. The analysis also considered the conceptual boundaries of academic FoMO in relation to adjacent constructs, to clarify its specific referent and reduce the risk of conceptual overlap.

## 3 Conceptual Definitions

To clarify academic FoMO with conceptual precision and empirical utility, it is necessary to define its core terms and distinguish them from adjacent constructs. The following definitions reflect the interdisciplinary foundations of the analysis and support clearer boundary specification.

### 3.1 Academic FoMO

Academic FoMO refers to a persistent, anxiety‐laden apprehension that one may be excluded from valuable academic opportunities, such as publications, collaborations, grants, invitations, or professional recognition.

Consistent with the original FoMO formulation, the core of the construct lies in the fear of missing professionally relevant opportunities from which one is or may be absent. Unlike general FoMO, which has often been studied in relation to social or digital experiences, academic FoMO is embedded in professional identity, institutional evaluation, and competitive visibility norms. It emerges in response to unmet psychological needs, perceived exclusion, and systemic pressures to remain visible and responsive. Academic FoMO is therefore conceptualized here as a dynamic and context‐sensitive process rather than a stable personality trait. In this framework, emotional discomfort, overengagement, and digital hypervigilance are treated as prototypical manifestations of academic FoMO, rather than as separate definitional components of the construct [1, 2].

#### 3.1.1 Overengagement

Overengagement describes a behavioral pattern of excessive, often misaligned academic activity driven by fear rather than interest. It includes constant monitoring of calls for papers, compulsive participation in low‐value collaborations, and pressure to maintain digital presence on academic platforms. Overengagement is not the result of ambition alone, but a response to perceived scarcity, visibility pressure, and comparison with peers. It often leads to emotional exhaustion, reduced motivation, and detachment from core academic values [7, 16].

#### 3.1.2 Motivational Frustration

Motivational frustration refers to the chronic frustration of the basic psychological needs for autonomy, competence, and relatedness, as described by SDT. In academic settings, these needs may be frustrated through distinct but interrelated pathways. Autonomy is undermined when researchers experience limited control over their research agenda, unstable contracts, dependence on senior gatekeepers, or strong pressures to align their activity with externally imposed metrics and expectations. Competence is frustrated when feedback is scarce or ambiguous, evaluative criteria are opaque, recognition is unstable, or one's work is repeatedly experienced as insufficient in relation to institutional standards. Relatedness is frustrated when scholars feel excluded from mentorship, collaboration, collegial exchange, or informal academic networks that confer belonging and professional legitimacy.

From this perspective, motivational frustration is not a generic background condition, but a central explanatory mechanism through which academic environments become psychologically consequential. When autonomy, competence, and relatedness are chronically frustrated, scholars may become more vigilant toward signs of exclusion, more dependent on external validation, and more likely to engage in compensatory monitoring and overengagement. In this sense, academic FoMO is conceptualized as one possible response to the repeated experience that one's academic participation is insufficiently agentic, insufficiently recognized, or insufficiently embedded in valued professional relationships [2, 3, 17, 18, 19].

#### 3.1.3 Visibility Pressure

Visibility pressure refers to the structural and cultural expectations within academia that individuals must remain visible, productive, and responsive to be considered successful. These expectations are reinforced through competitive metrics (e.g., citations, *h*‐index), social media exposure, and real‐time dissemination of academic achievements. Visibility pressure fuels the perception that absence equals irrelevance, thereby intensifying the drive to engage in FoMO‐related behaviors [12, 20].

## 4 Antecedents of Academic FoMO

**FIGURE 1:**
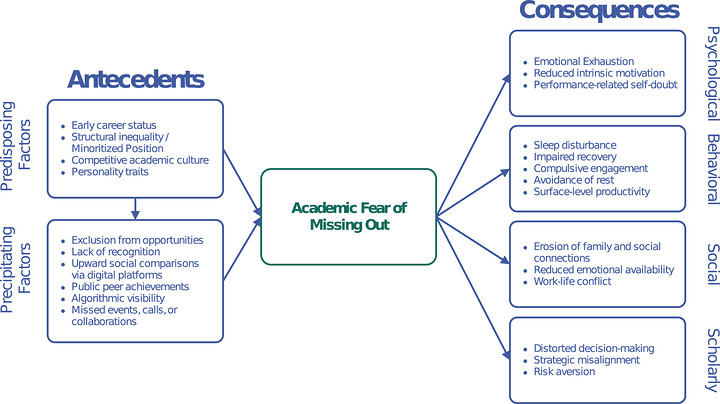
Antecedents, core concepts and consequences of academic FoMO.

The antecedents retained in the present model were selected because they recur across the FoMO literature and directly increase vulnerability to the core apprehension of missing academically valued opportunities. They are organized here into predisposing and precipitating factors (Figure 1) to distinguish longer standing vulnerabilities from situational activators [15].

### 4.1 Predisposing Factors

Predisposing factors include demographic, professional, and structural conditions that increase vulnerability to academic FoMO insofar as they are likely to chronically frustrate one or more basic psychological needs, or to heighten sensitivity to such frustration. For this reason, susceptibility to academic FoMO should not be attributed to age per se, but more precisely to early‐career and structurally precarious academic positions. Early‐career researchers, for instance, often navigate academic environments characterized by temporary contracts, unclear career trajectories, dependence on external evaluation, and limited access to institutional power, stable mentorship, and high‐value networks [21]. Such conditions may frustrate autonomy by reducing control over one's trajectory and priorities, competence by rendering evaluation and recognition uncertain, and relatedness by weakening durable inclusion in academic communities. In this sense, career‐stage vulnerability is theoretically relevant not because it represents a demographic category in itself, but because it repeatedly exposes scholars to need‐frustrating environments and to increased dependence on external validation [11]. A similar logic applies to gendered and minoritized positions in academia. Their relevance does not lie in demographic status alone, but in the greater likelihood of encountering exclusionary climates, unequal access to mentorship, funding, and recognition, and weaker integration into informal networks of sponsorship and belonging. These conditions may frustrate relatedness by undermining inclusion, competence by weakening affirmation and evaluative recognition, and, in some contexts, autonomy by constraining voice, participation, and room for self‐directed academic agency. Institutional cultures that promote hyperproductivity, individualism, and competitive comparison may further amplify these vulnerabilities by making external validation more salient and absence more threatening [10]. When academic value is narrowly defined through metrics and continuous output, individuals may internalize visibility norms, leading to anticipatory anxiety and performance‐driven engagement [20, 22].

Among the individual‐level predisposing factors, the broader empirical literature suggests that FoMO is not randomly distributed across persons but is more likely to emerge in the presence of identifiable dispositional vulnerabilities. A recent meta‐analysis found FoMO to be positively associated with neuroticism and negatively associated with conscientiousness, supporting the view that emotional instability and weaker self‐regulatory tendencies may increase susceptibility to FoMO‐related concerns [23]. In addition, more recent empirical work indicates that social comparison is a particularly strong proximal contributor to FoMO, while loneliness and perfectionism also make independent contributions [24]. In academic contexts, these patterns are especially relevant because highly evaluative environments intensify comparison‐based self‐appraisal and socially prescribed standards of performance. These dispositions are therefore best understood not as direct substitutes for contextual mechanisms, but as factors that may amplify reactivity to need frustration, perceived exclusion, and visibility‐based comparison.

### 4.2 Precipitating Factors

Precipitating factors refer to situational conditions that activate FoMO in already vulnerable contexts. These include perceived exclusion from academic opportunities, lack of recognition, and heightened exposure to others’ achievements through digital platforms. The visibility of peer success—publications, funding, invitations—can evoke upward comparisons and emotional responses such as anxiety, guilt, or inadequacy. Real‐time dissemination of professional milestones via platforms like LinkedIn, ResearchGate, and X increases the frequency and salience of these comparisons, especially for those with limited social capital or institutional support [12]. This can lead to compulsive engagement behaviors, including constant email checking, opportunistic participation in events, repeated checking of Scopus or Web of Science profiles and immediate responsiveness to institutional communications [7]. Academic evaluation systems grounded in performance indicators and publish‐or‐perish logics are likely to intensify these dynamics by framing nonparticipation in key opportunities as a potential professional liability; similarly, career insecurity has been shown to predict both informational and relational FoMO in work settings [10, 20, 25]. Under these conditions, even small perceived omissions, such as not being included in a collaborative project or missing a call for papers, can act as catalysts for academic FoMO [9]. In summary, academic FoMO is most likely to emerge at the intersection of psychological need frustration, structural precarity, and intensified social comparison, particularly in environments where academic value is tightly linked to visibility and continuous productivity.

A further precipitating condition concerns the perceived cost of leaving the academic system. Academic FoMO may be intensified when scholars strongly identify with the academic role, have invested substantially in academic credentials and networks, or perceive limited alternative career prospects. This may be especially relevant for early‐career researchers, whose well‐being and motivation are shaped by the satisfaction or frustration of autonomy, competence, and relatedness, and whose career development often involves uncertainty around available opportunities, institutional support, and future trajectories [17, 26]. Under these circumstances, missed opportunities may be experienced not simply as isolated setbacks, but as signals of possible exclusion from a career path that remains central to one's identity or appears difficult to replace.

It is important to emphasize that precipitating factors may occur as isolated incidents or clusters of events that interact with predisposing vulnerabilities. Their impact is particularly acute when they coexist with structural or psychological fragilities. For example, an early‐career researcher with limited mentorship may generally manage the pressures of academic life. However, being repeatedly excluded from collaborative projects or ignored in funding decisions can compound feelings of irrelevance and trigger anxiety‐driven overengagement. Similarly, a researcher from a minoritized background who typically maintains professional stability may experience an intensified fear of being left behind when exposed to a sudden wave of peer achievements shared online. In such cases, the intersection of institutional dynamics, social comparison, and identity‐based exclusion can significantly increase the likelihood of academic FoMO.

## 5 Defining Attributes and Process

Academic FoMO is best understood as a motivational–emotional response pattern shaped by environmental triggers, social norms, and psychological need frustration. Its core psychological nucleus is the apprehension of missing professionally relevant opportunities, recognition, or inclusion. In academic environments, this apprehension unfolds through a recurrent set of emotional, behavioral, and motivational manifestations, particularly digital hypervigilance, overengagement, controlled regulation, and performance‐driven relational anxiety. These elements interact dynamically over time, forming a self‐reinforcing and context‐sensitive process. Table 1 summarizes the main defining attributes of the construct.

**TABLE 1:** Attributes and processes of academic fear of missing out.

Component	Description	Indicative manifestations in academic contexts	Conceptual role
Apprehension of exclusion	Persistent concern about missing valuable academic opportunities, recognition, or inclusion.	Fear of not being invited into collaborations, missing calls, being overlooked for grants, authorship, or visibility.	Core psychological nucleus.
Digital hypervigilance	Continuous monitoring of opportunities and signals of inclusion or exclusion.	Frequent checking of email, newsletters, social media, academic platforms, calls, rankings, or profiles.	Behavioral expression that maintains the FoMO cycle.
Overengagement	Excessive involvement in activities not fully aligned with one's priorities.	Accepting low‐value collaborations, attending too many events, immediate responsiveness, difficulty saying no.	Compensatory response to perceived exclusion or invisibility.
Controlled regulation/motivational shift	Shift from autonomous engagement toward externally driven, visibility‐oriented regulation.	Prioritizing visible or strategically rewarding activities over meaningful or coherent academic goals.	Motivational mechanism reshaping academic behavior.
Performance‐driven relational anxiety	Distress related to exclusion from visible or prestigious academic spaces.	Anxiety when peers are invited, funded, cited, or included; excessive signaling to maintain relevance.	Relational‐emotional dimension tied to belonging, comparison, and recognition.

These elements distinguish academic FoMO from adjacent constructs such as impostor syndrome or generalized overcommitment. Whereas those patterns are more strongly centered on self‐doubt or enduring work style, academic FoMO is specifically characterized by fear of exclusion from valued opportunity structures within highly visible and competitive academic environments.

Another relevant component in the process of academic FoMO is the perceived level of self‐efficacy, defined as individuals’ beliefs in their capacity to execute behaviors necessary to produce specific performance attainments [27]. Scholars with higher levels of self‐efficacy are more likely to interpret exclusion or uncertainty as manageable challenges rather than threats to their academic identity. Conversely, low self‐efficacy can intensify emotional discomfort, amplify perceived exclusion, and fuel compulsive engagement. Based on existing evidence [28], academic self‐efficacy may act as both a mediator (by influencing how visibility pressures are translated into FoMO‐related responses) and as a moderator, buffering the negative psychological and behavioral consequences of academic FoMO. Its dual role highlights the importance of context‐specific beliefs in shaping vulnerability and resilience in competitive academic environments.

### 5.1 Illustrative Cases

**FIGURE 2:**
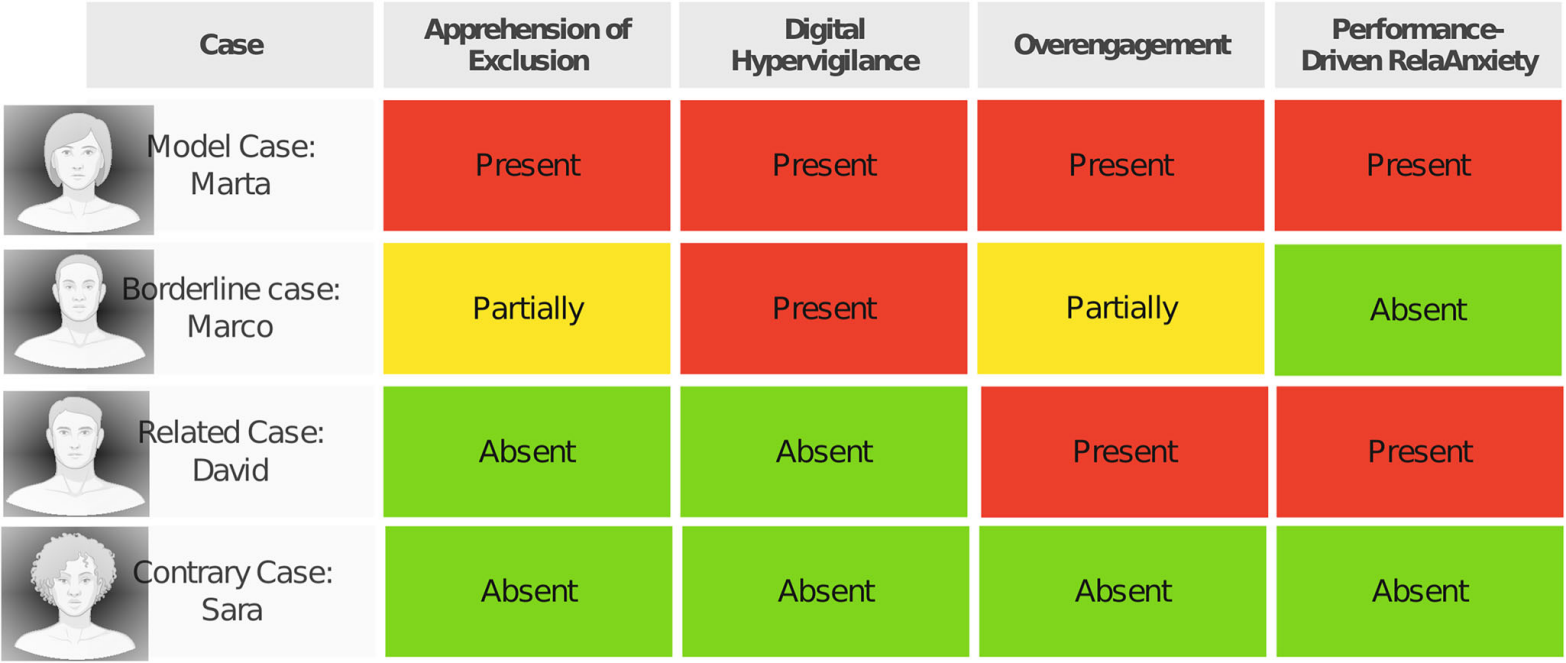
The presence, partial presence, or absence of the defining attributes of academic FoMO across four illustrative cases. The model case (Marta) represents the full expression of the construct, with all defining attributes clearly present: apprehension of exclusion from academically valued opportunities, digital hypervigilance, overengagement, and performance‐driven anxiety. The borderline case (Marco) shows only a partial fit: some behaviors resemble academic FoMO, particularly monitoring and selective overinvolvement, but these remain largely strategic rather than fear‐driven, and the core apprehension of exclusion is not fully developed. The related case (David) illustrates an adjacent but distinct pattern: distress and overengagement are present, yet they arise mainly from self‐doubt and perceived inadequacy rather than fear of missing out, and digital hypervigilance is absent. The contrary case (Sara) shows the absence of the defining attributes and therefore does not reflect academic FoMO. Together, these cases help clarify the conceptual boundaries of the construct and distinguish it from related forms of academic strain or involvement.

To complement the attribute‐based comparison shown in Figure 2, brief illustrative cases are presented to show how the defining attributes of academic FoMO may be fully present, partially present, or absent.

- Model case (Marta). Marta is an early‐career researcher who persistently fears being excluded from valuable academic opportunities and responds with frequent checking, overengagement, and anxiety‐driven efforts to remain visible.
- Borderline case (Marco). Marco closely monitors opportunities and sometimes overcommits, but his behavior is mainly strategic and does not reflect a persistent fear of exclusion or marked relational anxiety.
- Related case (David). David experiences academic distress and overengagement, but these are driven primarily by self‐doubt and perceived inadequacy rather than by fear of missing professional opportunities.
- Contrary case (Sara). Sara engages selectively in academic life, does not fear being left behind, and does not display the defining attributes of academic FoMO.

## 6 Consequences

At the psychological level, the broader FoMO literature already provides a meaningful empirical basis for several of the outcomes proposed here. Experience‐sampling and survey studies have shown that more frequent FoMO experiences are associated with greater stress, negative affect, fatigue, physical symptoms, and lower sleep. In occupational settings, workplace FoMO has been found to predict burnout and message‐checking behavior, while newer longitudinal evidence suggests that relational FoMO at work is associated with greater irritation and perceived stress. On this basis, academic FoMO is conceptualized here not as an entirely speculative phenomenon, but as an academic variant of an already documented strain pattern whose specific manifestations remain to be empirically tested in research environments [6, 7, 25].

At the organizational level, widespread academic FoMO may contribute to the normalization of unsustainable work cultures. Institutions that implicitly reward overexposure, hyperavailability, and digital visibility risk fostering environments where rest, selectivity, and silence are perceived as professional risks. This cultural climate may reinforce presenteeism, reduce psychological safety, and perpetuate cycles of burnout, especially among early‐career or structurally marginalized scholars [22].

A less documented consequence of academic FoMO concerns spillover into family and social life. At present, direct FoMO evidence on this issue remains limited, particularly in academic populations. However, emerging workplace research suggests that FoMO at work may extend beyond formal working hours through after‐work smartphone use and work–family conflict, thereby affecting broader psychological well‐being [29, 30]. In the present framework, we therefore retain relational spillover as a downstream pathway of interest, while explicitly treating it as a proposition requiring direct empirical verification rather than as an already established outcome.

More direct empirical support exists for recovery‐related outcomes. FoMO has been linked to shorter sleep duration and poorer sleep through nighttime social media use and heightened presleep cognitive arousal, and adult data further suggest that FoMO may indirectly predict problematic sleep through compulsive social media use and poor sleep hygiene. For this reason, in the present analysis we specify impaired recovery, evening cognitive activation, and sleep disturbance as evidence‐informed consequences of academic FoMO, while recognizing that the academic form of these processes still requires dedicated empirical testing [31, 32].

Moreover, academic FoMO has distorting effects on scholarly decision‐making. Researchers may prioritize visibility‐oriented actions—such as frequent but superficial publications, marginal coauthorships, or redundant participation in conferences—at the expense of depth, coherence, and long‐term contribution [33]. The pressure to appear active and connected may also inhibit risk‐taking, reduce intellectual creativity, and diminish authentic engagement with complex or controversial research topics. In the long run, these patterns can erode the quality of academic knowledge production and undermine collective trust within scholarly communities.

## 7 Assumptions and Propositions

### 7.1 Assumptions

The conceptual framework developed through this analysis is based on the following assumptions:

1. Academic environments are not neutral. Contemporary academic institutions are shaped by cultures of competition, productivity, and visibility, which influence scholars’ motivation, emotions, and behaviors.
2. Psychological needs are essential for academic well‐being. The satisfaction of autonomy, competence, and relatedness is fundamental for self‐regulation. When these needs are chronically frustrated, individuals may engage in compensatory behaviors such as overcommitment or digital hypervigilance.
3. Social comparison and visibility norms are structurally embedded. Academic systems that emphasize performance metrics, digital exposure, and constant productivity increase the salience of peer comparisons and the fear of being left behind.
4. Academic FoMO is a dynamic, context‐sensitive process. It is not a fixed personality trait but a motivational–emotional response that fluctuates according to perceived opportunities, threats, and social cues.
5. Institutional and individual factors interact. Academic FoMO emerges through the interplay of structural conditions (e.g., precarity, evaluation systems) and personal traits (e.g., perfectionism, self‐doubt).

### 7.2 Propositions

The following testable propositions arise from the present conceptual analysis of academic FoMO:

1. The interaction of predisposing and precipitating factors increases the likelihood of experiencing academic FoMO in researchers.
2. Academic FoMO is positively associated with psychological strain, including stress, emotional exhaustion, and reduced well‐being.
3. Academic FoMO is associated with maladaptive behavioral patterns, including compulsive engagement, excessive checking, and sleep/recovery difficulties.
4. Academic self‐efficacy mediates the relationship between visibility pressure and academic FoMO, such that lower perceived self‐efficacy in managing academic expectations increases susceptibility to FoMO‐related cognitions and behaviors.
5. Academic self‐efficacy moderates the relationship between academic FoMO and its psychological consequences, such that higher self‐efficacy buffers the negative impact of FoMO on stress, emotional exhaustion, and overengagement.
6. Maladaptive perfectionism, particularly socially prescribed perfectionism, is a significant predictor of academic FoMO in competitive academic settings.
7. Strong identification with the academic role and limited perceived alternative career prospects increase the likelihood that academic FoMO contributes to persistence in academia despite psychological strain.

## 8 Implications for Empirical Refinement

The concept analysis presented in this paper provides a foundation for future empirical refinement of academic FoMO. Although the concept is grounded in established motivational, organizational, and FoMO research, its boundaries, empirical referents, and discriminant validity remain to be tested across academic populations and settings. Future studies should therefore move from conceptual clarification to systematic operationalization.

A first priority is to identify empirical referents capable of representing the defining attributes of academic FoMO in observable or measurable form. Building on these referents, empirical studies are needed to develop psychometric instruments that accurately capture the specific features of academic FoMO. Existing FoMO scales [1] may offer a starting point but require revision to reflect the academic domain. A content‐validation process involving expert researchers, early‐career academics, and psychologists could ensure contextual relevance and construct validity.

Second, longitudinal and mixed‐methods research designs are particularly suited to explore how academic FoMO evolves over time, interacts with institutional environments, and affects well‐being and productivity. For instance, a prospective cohort study could track early‐career researchers over 12–24 months to investigate how fluctuations in perceived academic exclusion predict burnout, disengagement, or changes in publication patterns. Qualitative studies using in‐depth interviews or digital diaries may also uncover the nuanced emotional and behavioral dimensions of academic FoMO.

Third, future research should focus on high‐risk academic subgroups where academic FoMO may be particularly salient. These include PhD students navigating identity formation and institutional uncertainty; precarious researchers with fixed‐term contracts or fellowships; women and minoritized scholars facing structural barriers to visibility and inclusion; researchers working in hypercompetitive, under‐resourced, or internationalized environments.

Additionally, studies could investigate protective and moderating factors such as emotional self‐efficacy, mentorship quality, institutional culture, and digital literacy. Exploring these variables may help identify intervention targets to mitigate the adverse consequences of academic FoMO.

Finally, cross‐cultural validation of the construct would extend its generalizability, as academic norms, competitive dynamics, and institutional demands vary significantly across national systems. Comparative studies across countries or academic disciplines could provide insight into how context shapes the expression and impact of academic FoMO.

A critical empirical task for future research will be to assess whether academic FoMO demonstrates adequate discriminant validity relative to conceptually adjacent constructs. In line with recommendations on construct proliferation [14], future validation studies should examine whether academic FoMO can be empirically distinguished from related constructs using multitrait and multivariate approaches.

In sum, the present concept analysis opens the door to a research agenda that is both theoretically grounded and practically relevant. Future empirical efforts should aim not only to measure and model academic FoMO but also to refine its boundaries, test its distinctiveness, and inform strategies for promoting sustainable academic engagement, inclusive career development, and institutional well‐being.

## Author Contributions

Mattia Bozzetti: conceptualization, writing – original draft, project administration, supervision. Alessio Lo Cascio: conceptualization, writing – original draft, project administration, supervision. Daniele Napolitano: conceptualization, writing – original draft, project administration, supervision.

## Conflicts of Interest

The authors declare no conflicts of interest.
